# Assessment of Multidimensional Biosafety Practices in Clinical Dental Training: A Cross‐Sectional Study

**DOI:** 10.1002/hsr2.72838

**Published:** 2026-09-27

**Authors:** Abdullah F. Alshammari, Ahmed A. Madfa, Basel R. Alkhalil, Dhay M. Alazaima, Hamdan A. Alshammari, Asma Z. Alshammari, Saleh M. Aloqla, Sattam S. Alshammari

**Affiliations:** ^1^ Department of Basic Dental and Medical Science, College of Dentistry University of Ha'il Ha'il Kingdom of Saudi Arabia; ^2^ Department of Restorative Dental Science, College of Dentistry University of Ha'il Ha'il Kingdom of Saudi Arabia; ^3^ College of Dentistry University of Ha'il Ha'il Kingdom of Saudi Arabia; ^4^ Department of Preventive Dental Science, College of Dentistry University of Ha'il Ha'il Kingdom of Saudi Arabia

**Keywords:** biosafety, clinical training, dental education, infection control, personal protective practices, waste management

## Abstract

**Background and Aims:**

Biosafety and infection prevention are essential components of dental clinical training, yet adherence to recommended practices among undergraduate dental students remains variable. This study evaluates biosafety and infection prevention practices among undergraduate dental students at the University of Ha'il College of Dentistry.

**Methods:**

This cross‐sectional analytical study was conducted among 99 undergraduate dental students undertaking clinical training at the College of Dentistry, University of Hail, Saudi Arabia. A standardized self‐administered questionnaire was used to evaluate five biosafety domains: Personal Protective Protocol (PPP), Dental Unit Management Protocol (DUMP), instrument management Protocol (IMP), Waste Management Protocol (WMP), and Ergonomics Protocol (EP). Scores were standardized to reflect levels of compliance. Nonparametric tests (Kruskal–Wallis) were used to compare biosafety practices across clinical departments, and Spearman's correlation analysis (*ρ*) was used to examine relationships among biosafety domains, with effect sizes reported as correlation coefficients.

**Results:**

Students demonstrated moderate to high adherence across most domains, with the highest compliance in DUMP (mean = 0.78, SD = 0.22) and WMP (mean = 0.78, SD = 0.23). Greater variability was observed in PPP (mean = 0.67, SD = 0.29) and EP (mean = 0.74, SD = 0.33). Statistically significant interdepartmental differences were observed only for DUMP (*p* = 0.045), with higher adherence in technique‐intensive departments. Positive correlations were observed between DUMP and IMP (*ρ* = 0.205, *p* = 0.042) and between IMP and WMP (*ρ* = 0.264, *p* = 0.008), indicating weak associations between these domains. However, the strength of these correlations is limited. PPP showed no statistically significant correlations with the other domains, suggesting largely independent patterns of behavior.

**Conclusion:**

In general, undergraduate dental students exhibited satisfactory biosafety practices, although notable gaps persist in personal protective behaviors and environmental protection. The observed interdomain correlations highlight the interconnected nature of biosafety compliance. Targeted, department‐specific educational interventions and continuous monitoring may strengthen biosafety culture and improve consistency across clinical training environments.

## 1 Introduction

Biosafety and infection prevention are fundamental pillars of dental education and clinical practice, protecting patients, dental students, faculty members, and auxiliary staff from exposure to infectious agents and other occupational hazards. Due to frequent interaction between patients and operators, exposure to blood and saliva, and the extensive production of aerosols and spatter during surgical operations, dental clinical settings are susceptible to cross‐infection [1, 2, 3]. Therefore, rigorous adherence to biosafety protocols is not just a professional need but also a fundamental skill that has to be ingrained throughout undergraduate dentistry education.

Comprehensive infection control and biosafety recommendations for dental settings have been developed by internationally renowned organizations, including the World Health Organization (WHO), the Centers for Disease Control and Prevention (CDC), and major dental associations [4, 5, 6]. These recommendations cover a wide range of topics, including waste management, environmental protection (EP), instrument processing and sterilization, hand hygiene, and personal protective measures. However, several studies have shown that adherence to these norms is suboptimal, especially among dental students and recently graduated practitioners [7, 8, 9]. The non‐adherence rates raise concerns regarding the efficacy of biosafety education and its application in routine clinical practice.

Given that professional behaviors and attitudes are actively formed during the clinical training years, dental students represent a crucial demographic for biosafety evaluation [10, 11]. The workload, departmental culture, level of supervision, and clinical learning environment all significantly impact the extent to which students adhere to infection control procedures. Furthermore, different dental specializations have varying clinical processes. Compared to other specialities, departments such as Endodontics, Periodontics, and Oral and Maxillofacial Surgery may have higher perceived and actual infection risks due to their invasive procedures and increased exposure to blood, saliva, and aerosols [12, 13, 14]. These variations may lead to different biosafety procedures across departments, underscoring the importance of department‐based assessments.

Biosafety practice is a multifaceted construct rather than a single behavior, according to modern infection control methods [15]. The total degree of biosafety compliance is determined by several factors, including personal protective practices (PPP), disinfection and sterilization behaviors, instrument management practices (IMP), waste management practices (WMP), and EP. A more detailed view of the advantages and disadvantages of clinical training programs is made possible by evaluating these elements independently. To promote integrated educational initiatives, it may also be possible to determine whether adherence in one area positively affects compliance in others by examining the links between these components [16].

The surge in dental education in Saudi Arabia has coincided with an enhanced focus on infection control, patient safety, and quality assurance in educational institutions [17]. Future dental professionals in the Hail region and the Kingdom as a whole receive a substantial portion of their training from the University of Hail's College of Dentistry. Evaluating biosafety measures across departments is especially important given the college's diverse clinical departments and the different procedural risk levels students face. In addition to ensuring adherence to national and international standards, this kind of evaluation is crucial for fostering a consistent safety culture among students when they enter the workforce.

Despite the widely acknowledged significance of biosafety, there is a dearth of published data that thoroughly assesses the components of biosafety practices across dental departments in Saudi dental schools, especially in newly established academic institutions. Furthermore, few studies have examined the relationships among different biosafety practice domains to determine whether advancements in one area may lead to improved compliance across the others. Therefore, this study aims to investigate the distribution of biosafety practice scores among dental students at the University of Hail's College of Dentistry, compare these scores across clinical departments, and assess the relationships between the main components of biosafety practice.

## 2 Methods

Ethical approval was obtained from the Institutional Review Board at the University of Hail, in accordance with ethical research guidelines (Approval No. H‐2025‐612). All participants’ data were fully anonymized to ensure confidentiality and privacy. Written informed consent was obtained from all participants before participation, and all collected data were used only for research purposes.

This study was a cross‐sectional analytical design conducted at the College of Dentistry, University of Hail, Saudi Arabia. Undergraduate dental students receive supervised clinical training in several departments within the college, including Endodontics, Pediatric Dentistry, Operative Dentistry, Comprehensive Clinical Care, Oral and Maxillofacial Surgery, Prosthodontics, Orthodontics, and Periodontics. The study aimed to assess biosafety practices, compare them across clinical departments, and examine relationships among different biosafety domains.

The study population included undergraduate dental students in the clinical phase of the dentistry program, usually in the fourth, fifth, and sixth academic years. Preclinical students, interns, postgraduate trainees, and students who did not complete the questionnaire were excluded. Only students who were actively participating in clinical sessions during the study period and who agreed to participate were included.

Convenience sampling was used. All eligible students present during the data collection period were invited to participate. Undergraduate students in the fourth, fifth, and sixth years who were actively involved in clinical sessions and provided informed consent were included, while preclinical students, interns, postgraduate trainees, absent students, and those who did not complete the questionnaire were excluded. This approach followed the normal clinical training distribution and allowed representation from different departments.

A structured, self‐administered questionnaire was used for data collection. It was developed based on biosafety and infection control guidelines from the World Health Organization (WHO) and the Centers for Disease Control and Prevention (CDC), and supported by previously published studies. The questionnaire assessed biosafety practices across five domains: Personal Protection Protocol (PPP, 5 items), Dental Unit Management Protocol (DUMP, 5 items), Instrument Management Protocol (IMP, 3 items), Waste Management Protocol (WMP, 5 items), and Ergonomics Protocol (EP, 3 items).

The questionnaire included structured items with fixed response options to allow objective measurement of compliance with biosafety standards. For each domain, responses were scored and standardized, with higher scores indicating better compliance. EP scores reflected combined behavioral practices and showed wider variation, while PPP, DUMP, IMP, and WMP scores were standardized to a 0–1 scale. Mean scores were calculated for each domain to evaluate overall compliance. The full questionnaire is provided in Supporting Information S1.

After explaining the study purpose and confirming voluntary participation, questionnaires were given to students during clinical sessions following institutional consent procedures. All participants gave consent before participation. Confidentiality and anonymity were strictly maintained throughout the study. Completed questionnaires were collected immediately to reduce missing data and response bias.

The instrument consisted of 21 items across five domains: Personal Protection Protocol (PPP, 5 items), Dental Unit Management Protocol (DUMP, 5 items), Instrument Management Protocol (IMP, 3 items), Waste Management Protocol (WMP, 5 items), and Ergonomics Protocol (EP, 3 items). Each item was scored as 1 if the correct procedure was followed and 0 if not followed. For each domain, item scores were summed and divided by the number of items to create a standardized compliance score. PPP, DUMP, and WMP were divided by 5, while IMP and EP were divided by 3. All scores were entered into Microsoft Excel for further statistical analysis.

Content validity of the questionnaire was assessed by experts in dental research and infection control. A pilot study was performed on a small group of dentists who were not included in the final sample. Their feedback was used to improve the clarity and wording of the questionnaire. Reliability was tested using Cronbach's alpha, which showed acceptable to good internal consistency.

All statistical analyses were performed using IBM SPSS version 23.0 (IBM Corp., Armonk, NY, USA). Descriptive statistics included mean, standard deviation (SD), median, interquartile range (IQR), minimum, and maximum values. SD and IQR were used to describe variability in the data. Normality of data was checked using visual inspection and statistical tests; therefore, non‐parametric methods were used. The Kruskal–Wallis test (H statistic) was used for planned comparisons of biosafety scores across clinical departments. Spearman's rank correlation coefficient (*ρ*) was used for exploratory analysis of relationships between biosafety domains. Results were interpreted using both *p*‐values and effect size direction and strength, following recommended statistical reporting guidelines. All statistical terms (H, ρ, SD, IQR) were defined at first use. A two‐sided *p*‐value < 0.05 was considered statistically significant, and *p* < 0.01 was considered highly significant. Interpretation of results focused not only on statistical significance but also on the magnitude and practical relevance of findings, in line with recommended clinical research reporting standards.

## 3 Results

All 99 participants included in the study had complete data for the variables of interest, with no missing values. Specifically, information was available for each participant regarding their clinical department and scores for PPP, DUMP, IMP, WMP, and EP. Therefore, the full dataset of 99 cases was used in all subsequent analyses.

### 3.1 Overall Descriptive Characteristics of Biosafety Practice Domains

The assessment of multidimensional biosafety practices among clinical dental students revealed generally moderate to high levels of adherence across the evaluated domains. Descriptive analysis demonstrated that PPP yielded a mean score of 0.67 (SD = 0.29), with scores spanning from 0.20 to the maximum possible value of 1.00. This range indicates noticeable variability in the consistent application of personal protective measures among participants.

DUMP had one of the highest mean scores across all domains, averaging 0.78 (SD = 0.22). Scores ranged from 0.20 to 1.00, reflecting relatively stable compliance with surface disinfection, unit decontamination, and related biosafety procedures in clinical environments. IMP also showed favorable adherence levels, with a mean score of 0.74 (SD = 0.27) and a narrower distribution of scores, suggesting generally consistent handling, processing, and management of dental instruments.

WMP exhibited a mean score of 0.78 (SD = 0.23), comparable to that of DUMP, with observed values ranging from 0.20 to 1.00. This indicates a generally high level of compliance with waste segregation, disposal, and related biosafety procedures. EP scores showed greater variability than other domains, with a mean score of 0.74 (SD = 0.33) and an observed range of 0–2, highlighting greater heterogeneity in behaviors related to environmental safety and clinic surroundings.

A comprehensive summary of central tendency and dispersion measures for all biosafety domains is provided in Table 1.

**Table 1:** Descriptive overview of department and biosafety practice scores.

	Mean	Std. deviation	Minimum	Maximum
Department	4.3131	1.96212	1	8
PPP score	0.6727	0.28636	0.2	1
DUBP score	0.7798	0.21852	0.2	1
IMP score	0.7392	0.27355	0.3	1
WMP score	0.7798	0.22767	0.2	1
EP score	0.7385	0.32551	0	2

### 3.2 Departmental Distribution of PPP

Detailed interdepartmental comparisons for all biosafety domains are presented in Table 2. PPP scores were compared across clinical departments using the Kruskal–Wallis test, with results showing no statistically meaningful differences in distribution across groups (*H* = 7.11, *p* = 0.119). Although not statistically significant, descriptive effect patterns indicated higher mean ranks for PPP in Periodontics, followed by Endodontics and Oral and Maxillofacial Surgery, whereas lower mean ranks were observed in Comprehensive Clinical Care and Pediatric Dentistry. These findings suggest comparable levels of personal protective practice across departments despite observable rank variation.

**Table 2:** Comparison of biosafety practice scores across departments.

	Department	Mean rank	*p*‐value
PPP score	Endodontics	66.5	0.119
	Pediatric	48.85	
	Operative	52.86	
	CCC	37.19	
	Surgery	57.45	
	Prosthodontics	45.59	
	Orthodontic	50	
	Periodontics	71	
DUMP score	Endodontics	70.06	0.045
	Pediatric	50.88	
	Operative	46.5	
	CCC	41	
	Surgery	39.45	
	Prosthodontics	56.68	
	Orthodontic	65.1	
	Periodontics	33.5	
IMP score	Endodontics	53.88	0.426
	Pediatric	50.38	
	Operative	41.75	
	CCC	41.52	
	Surgery	47.91	
	Prosthodontics	60.15	
	Orthodontic	57.2	
	Periodontics	57.2	
VMP score	Endodontics	60.38	0.549
	Pediatric	44.62	
	Operative	39.43	
	CCC	52.57	
	Surgery	47.32	
	Prosthodontics	54.03	
	Orthodontic	58.65	
	Periodontics	41.1	
EP score	Endodontics	58.19	0.07
	Pediatric	38.88	
	Operative	37.82	
	CCC	63.1	
	Surgery	43.09	
	Prosthodontics	57.03	
	Orthodontic	46.45	
	Periodontics	43.3	

### 3.3 Departmental Differences in DUMP

Significant differences in DUMP were observed across clinical departments (*H* = 9.76, *p* = 0.045), indicating variability in disinfection‐related practices. Endodontics demonstrated the highest mean rank, followed by Orthodontics and Prosthodontics, suggesting stronger adherence to unit disinfection protocols in these departments. In contrast, lower mean ranks were observed in Periodontics and Comprehensive Clinical Care, indicating comparatively reduced compliance in these groups. These findings highlight meaningful interdepartmental differences in environmental and unit decontamination practices.

### 3.4 IMP Across Departments

No statistically significant differences were observed in IMP across departments (*H* = 3.90, *p* = 0.426). Mean ranks showed modest variation, with higher ranks in Prosthodontics, Orthodontics, and Periodontics, and lower ranks in Operative Dentistry and Comprehensive Clinical Care. These results suggest broadly consistent instrument handling and processing practices across departments.

### 3.5 WMP Across Departments

WMP also did not differ significantly across departments (*H* = 3.04, *p* = 0.549). Although Endodontics and Orthodontics demonstrated relatively higher mean ranks, and Operative Dentistry and Periodontics lower ranks, these differences were small and not indicative of meaningful variation. Overall, waste management practices appeared relatively uniform across clinical training settings.

### 3.6 EP Practices Across Departments

Differences in EP across departments did not reach statistical significance (*H* = 9.07, *p* = 0.070). Higher mean ranks were observed in Comprehensive Clinical Care and Endodontics, while lower ranks were seen in Operative Dentistry and Pediatric Dentistry. Despite observable variation, the effect was not strong enough to indicate consistent interdepartmental differences.

### 3.7 Correlation Analysis Between Biosafety Practice Domains

Spearman's rank correlation analysis (Table 3) was used to examine the relationships among biosafety domains. A weak but statistically significant positive correlation was found between DUMP and IMP (*ρ* = 0.205, *p* = 0.042), showing a slight association between disinfection practices and instrument management. A weak positive correlation was also observed between IMP and WMP (*ρ* = 0.264, *p* = 0.008), indicating a modest relationship between instrument processing and waste management practices; however, these correlations are small in strength. No statistically significant correlations were found between PPP and other domains, or between EP and the remaining biosafety measures, suggesting that these behaviors are mostly independent.

**Table 3:** Spearman's rank correlations among PPP, DUMP, IMP, WMP, and EP scores.

Variable pair	Spearman rank	Sig. (2‐tailed)
PPP–DUMP	0.186	0.065
PPP–IMP	−0.065	0.520
PPP–WMP	−0.022	0.830
PPP–EP	0.050	0.622
DUMP–IMP	0.205*	0.042
DUMP–WMP	0.089	0.379
DUMP–EP	0.156	0.123
IMP–WMP	0.264**	0.008
IMP–EP	0.100	0.324
WMP–EP	0.154	0.127

* *p* < 0.05

** *p* < 0.01.

## 4 Discussion

This study provides a thorough assessment of the multimodal biosafety measures used by undergraduate dentistry students at the University of Hail during their clinical rotations. Overall, the results show moderate to high levels of adherence in the majority of biosafety areas, with some interdepartmental variances and significant domain heterogeneity. These findings support the idea that biosafety compliance in dentistry education is complex and affected by both the clinical setting and individual behaviors.

The comparatively high mean ratings for WMP and DUMP indicate that students are usually well‐oriented toward visible, protocol‐driven infection control actions. Similar patterns have been observed in other research, in which adherence to trash disposal and surface disinfection was greater than to other infection control measures [7, 13, 17, 18, 19]. This may be because of strict institutional enforcement and regular monitoring. The comparatively uniform adherence across departments may be explained by the standardized nature of these procedures, which are often supported by checklists and faculty oversight.

PPP, on the other hand, had a lower mean score and more variability. This result is consistent with previous publications showing that dental students utilize personal protective equipment (PPE) inconsistently, especially masks and eye protection, even when they have a sufficient understanding of infection control concepts [8, 9, 11, 20]. The lack of statistically significant differences in PPP across departments suggests that individual attitudes, comfort, and perceived risk may have a greater impact on these behaviors than departmental procedures alone. According to studies, students are more likely to adhere to PPP during operations they perceive as very invasive or high‐risk [10, 21]. This suggests that risk perception significantly influences PPE compliance.

There were no statistically significant interdepartmental variations in the positive, generally consistent adherence to IMP. This uniformity may result from standardized instrument processing procedures and centralized sterilization rules commonly used in dental schools [15, 16]. Centralized sterilizing units have been shown to enhance compliance and decrease variability across clinical specialities in regional and global studies [14, 22]. These solutions strengthen consistent infection control guidelines and lessen the need for individual decisions.

DUMP demonstrated statistically significant interdepartmental variance, despite most biosafety domains not changing substantially across departments. Higher adherence was observed in departments such as Endodontics, which may be related to greater knowledge of the hazards of cross‐contamination associated with endodontic treatments, including exposure to blood, irrigants, and infected root canal instruments [12, 23]. On the other hand, lower mean rankings in departments such as Comprehensive Clinical Care could be due to increased workload, a broader procedural scope, and higher patient turnover, all of which have been linked to worse compliance in prior research [16, 24].

The ratings for EP procedures were the most widely distributed and did not differ considerably between departments. This variation implies that, compared with other aspects of infection control, formal training may place less emphasis on environmental biosafety behaviors, such as ventilation awareness, spill management, and maintenance of clinic surroundings. Similar findings from other studies indicate that environmental safety is often given less emphasis than PPE or sterilization procedures in biosafety education in dentistry [18, 25]. This result emphasizes the need for undergraduate programs to explicitly incorporate EP themes.

The correlation analysis suggests that biosafety behaviors are weakly related across different domains. A small positive association was observed between DUMP and IMP, indicating that students who follow surface disinfection practices may also slightly tend to handle instruments more appropriately. However, this relationship is weak and should not be interpreted as strong clustering behavior. Similarly, the weak association between IMP and WMP may reflect some shared procedural routines related to instrument processing and waste management, but the strength of these relationships is limited. Overall, these findings should be interpreted cautiously and do not strongly support a broad “culture of safety” across all domains [15, 26].

Interestingly, no significant correlation was found between personal protective behaviors and other biosafety categories. This result has been previously documented and may suggest that personal habits, discomfort, or perceived difficulty have a greater impact on PPE usage than systemic infection control procedures [11, 20, 27]. Instead of relying solely on legislation to close this gap, clinical instructors may need to implement targeted behavioral treatments, ongoing reinforcement, and role modeling.

The results of this study are in line with regional statistics from Saudi Arabia and surrounding nations, which indicate that dentistry students and healthcare professionals have acceptable but attainable levels of compliance with infection control [13, 14, 17, 28]. To ensure uniform biosafety requirements and long‐term compliance, department‐specific input and ongoing monitoring are crucial, given the Kingdom's surge in dental education.

Overall, the study shows that although undergraduate dentistry students typically adhere to biosafety standards, significant gaps remain in several areas. While targeted behavioral reinforcement is necessary for environmental and personal protective measures, centralized protocols and standardized systems facilitate consistent performance in areas such as waste management and instrument handling. Interdepartmental variations in disinfection procedures highlight the necessity for department‐specific monitoring and workflow modifications. Integrated training methods may improve overall compliance, as indicated by correlations across several biosafety areas. To ensure safe clinical dentistry practice, biosafety culture must be strengthened through ongoing monitoring, targeted education, and strong faculty role modeling.

The cross‐sectional nature of this study limits its ability to evaluate causal linkages or changes over time. Bias may have been introduced by relying on self‐reported questionnaires, and actual compliance may not align with stated practices due to the lack of observational verification. This study employed convenience sampling of all undergraduate students at the College of Dentistry, University of Hail. As a result, the findings may not be generalizable to other dental schools, and the sampling method may introduce potential selection bias. The use of self‐reported questionnaires may introduce social desirability bias, and reported behaviors may not fully align with actual practices observed in clinical settings. Furthermore, departmental variations, clinical burden, and faculty supervision were not adequately taken into consideration. Finally, cultural and contextual factors specific to the University of Hail and the Saudi Arabian dental education system may influence compliance behaviors, and these results may not directly translate to other educational or healthcare contexts. Despite these drawbacks, the study provides valuable insights into multifaceted biosafety procedures and identifies areas of dental education that require improvement.

## 5 Conclusion

The study found that undergraduate dental students at the University of Hail generally adhere well to biosafety practices, with strong compliance in disinfection, unit biosafety, and waste management. There was greater variation in EP practices and personal protective measures, indicating areas for improvement. While there was significant variety in disinfection techniques, there were very few interdepartmental disparities. Integrated training may improve overall compliance, as indicated by correlations across certain areas. Overall, the results highlight the necessity of continual observation, focused instruction, and reinforcement to promote a constant culture of safety in dentistry clinical training.

## Author Contributions

**Abdullah F. Alshammari:** conceptualization, investigation, funding acquisition, writing – original draft, writing – review and editing, visualization, validation, methodology, project administration, formal analysis, software, resources, supervision, data curation. **Ahmed A. Madfa:** writing – original draft, funding acquisition, investigation, conceptualization, writing – review and editing, visualization, validation, methodology, project administration, formal analysis, software, resources, supervision, data curation. **Basel R. Alkhalil:** methodology, visualization, writing – review and editing, data curation, formal analysis, investigation, writing – original draft. **Dhay M. Alazaima:** investigation, methodology, validation, formal analysis, writing – review and editing, writing – original draft. **Hamdan A. Alshammari:** investigation, writing – original draft, writing – review and editing, methodology, formal analysis. **Asma Z. Alshammari:** writing – original draft, investigation, methodology, writing – review and editing, formal analysis. **Saleh M. Aloqla:** investigation, writing – original draft, writing – review and editing, data curation, methodology. **Sattam S. Alshammari:** data collection, review and editing.

## Disclosure

The institutions used did not have any role in study design; collection, analysis, and interpretation of data; writing of the report; and the decision to submit the report for publication.

## Ethics Statement

Ethical approval was obtained from the Institutional Review Board at the University of Hail, in accordance with ethical research guidelines (Approval No. H‐2025‐612).

## Consent

The authors confirmed that informed consent was obtained from all subjects. The authors confirm that all methods were performed following the relevant guidelines and regulations, for example, the Declaration of Helsinki.

## Conflicts of Interest

The authors declare no conflicts of interest.

## Transparency Statement

The lead authors, Abdullah F. Alshammari and Ahmed A. Madfa, affirm that this article is an honest, accurate, and transparent account of the study being reported; that no important aspects of the study have been omitted; and that any discrepancies from the study as planned (and, if relevant, registered) have been explained.

## Supporting information


Supporting File

## Data Availability

The data that support the findings of this study are available on request from the corresponding author. The data are not publicly available due to privacy or ethical restrictions.
