# Supplementary material for: Assessment of Multidimensional Biosafety Practices in Clinical Dental Training: A Cross‐Sectional Study
Source: Health Sci Rep. 2026 Sep 27;9(10):e72838. doi: 10.1002/hsr2.72838 (PMC13617310; doi:10.1002/hsr2.72838)
Supplement: Supplementary file 1 [file HSR2-9-e72838-s001.pdf]

D1: Personal Protection Protocol (PPP)

		Endodontics	Pediatric	Operative	CCC	Surgery	Prosthodontics	Orthodontics	Overall
PPE	**Use of complete PPE by The operator**								
	**Use of PPE by assistants**								
	**Use of the complete PPE By clinic instructor**								
	**Use of clinical gloves**								
	**Use of polyethylene gloves**								
**Total of adherence**									

D2: Dental Unit Management Protocol (DUMP)

		Endodontics	Pediatric	Operative	CCC	Surgery	Prosthodontics	Orthodontics	Overall
DUMAP	**Use of plastic barriers**								
	**Surfaces disinfection**								
	**Use of nitrile gloves for cleaning**								

	**Reception of clean units**								
	**Presentation of clean units**								
**Total of adherence**									

D3: Instrument Management Protocol (IMP)

		Endodontics	Pediatric	Operative	CCC	Surgery	Prosthodontics	Orthodontics	Overall
IMP	**Use of sterile instruments**								
	**Use of sterile field on The worktable**								
	**Implementation of the instrument organization system**								
**Total of adherence**									

D4: Waste Management Protocol (WMP)

		Endodontics	Pediatric	Operative	CCC	Surgery	Prosthodontics	Orthodontics	Overall
WMP	**Waste separation**								
	**Proper disposal of PPE**								
	**Proper disposal of Infectious biological Hazardous waste**								
	**Proper disposal of paper/plastic**								

	**Proper disposal of sharp objects**								
**Total of adherence**									

D5: Ergonomics Protocol (EP)

		Endodontics	Pediatric	Operative	CCC	Surgery	Prosthodontics	Orthodontics	Overall
	**Correct position of the operator**								
	**Use of adequate workspaces**								
ERP	**Minimum use of push-ups and back extensions**								
**Total of adherence**									
